# TPA-023 attenuates subchronic phencyclidine-induced declarative and reversal learning deficits via GABA_A_ receptor agonist mechanism: possible therapeutic target for cognitive deficit in schizophrenia

**DOI:** 10.1038/s41386-018-0160-3

**Published:** 2018-07-23

**Authors:** Lakshmi Rajagopal, Mei Huang, Eric Michael, Sunoh Kwon, Herbert Y. Meltzer

**Affiliations:** Department of Psychiatry and Behavioral Sciences, Northwestern University Feinberg School of Medicine, Chicago, IL 60611 USA

## Abstract

GABAergic drugs are of interest for the treatment of anxiety, depression, bipolar disorder, pain, cognitive impairment associated with schizophrenia (CIAS), and other neuropsychiatric disorders. Some evidence suggests that TPA-023, (7-(1,1-dimethylethyl)-6-(2-ethyl-2H-1,2,4-triazol-3-ylmethoxy)-3-(2-fluorophenyl)-1,2,4-triazolo[4,3-b] pyridazine), a GABA_A_ α2,3 subtype-selective GABA_A_ partial agonist and α_1/5_ antagonist, and the neurosteroid, pregnenolone sulfate, a GABA_A_ antagonist, may improve CIAS in pilot clinical trials. The goal of this study was to investigate the effect of TPA-023 in mice after acute or subchronic (sc) treatment with the *N*-methyl-d-aspartate receptor (NMDAR) antagonist, phencyclidine (PCP), on novel object recognition (NOR), reversal learning (RL), and locomotor activity (LMA) in rodents. Acute TPA-023 significantly reversed scPCP-induced NOR and RL deficits. Co-administration of sub-effective dose (SED) TPA-023 with SEDs of the atypical antipsychotic drug, lurasidone, significantly potentiated the effect of TPA-023 in reversing the scPCP-induced NOR deficit. Further, scTPA-023 co-administration significantly *prevented* scPCP-induced NOR deficit for *5* weeks. Also, administration of TPA-023 for 7 days following scPCP reversed the NOR deficit for 1 week. However, TPA-023 did not blunt acute PCP-induced hyperactivity, suggesting lack of efficacy as a treatment for psychosis. Systemic TPA-023 significantly blocked lurasidone-induced increases in cortical acetylcholine, dopamine, and glutamate without affecting increases in norepinephrine and with minimal effect on basal release of these neurotransmitters. TPA-023 significantly inhibited PCP-induced cortical and striatal dopamine, serotonin, norepinephrine, and glutamate efflux. These results suggest that TPA-023 and other GABA_A_ agonists may be of benefit to treat CIAS.

## Introduction

Multiple GABAergic drugs have been investigated for the treatment of the cognitive impairment associated with schizophrenia (CIAS), positive and negative symptoms [1–3]. GABAergic stimulation of GABA_A_ and GABA_B_ receptors (Rs) is crucial to the maintenance of the excitation/inhibition (E/I) balance in the brain and for the entrainment of oscillatory activities required for normal cognition [4]. The abnormality in gamma synchronization associated with CIAS has been attributed to GABAergic dysfunction [5]. Furthermore, there is evidence of diminished GABA synthesis, gene expression, altered types of GABA_A_R subunits in cortex, altered levels of multiple GABA genes, and electrophysiological dysfunction in GABAergic interneurons in parvalbumin–immunoreactive (PV-IR) and somatostatin GABA interneurons in the cortex and hippocampus (HIP) [6–8]. Development of GABA-based treatments for CIAS are a high priority [6, 9].

Glutamate (Glu), as the major excitatory neurotransmitter in the brain, is crucial to maintaining a normal E/I balance. Together, GABA interneurons and pyramidal Glu neurons are the building blocks of neural circuits that underlie normal brain function. Among the most compelling evidence for the roles of Glu and GABA in schizophrenia is the ability of *N*-methyl-d-aspartate receptor (NMDAR) antagonists, e.g., phencyclidine (PCP) and ketamine, to produce symptoms of schizophrenia in normal controls and exacerbate psychosis and cognitive impairment in schizophrenia patients. For this reason, acute and especially subchronic (sc) administration of NMDAR antagonists such as PCP and MK-801 have been extensively studied as models of psychosis, negative symptoms, and CIAS [9–14]. ScPCP administration has been reported to produce change in cortical gene expression [15] that overlap with those that differ in postmortem brain tissue from patients with schizophrenia compared to normal controls [16]. Acute PCP administration has been reported to produce impairments in multiple types of cognitive function in rodents [14, 17–19]. PCP and MK-801 significantly increased locomotor activity (LMA) in rodents, an effect which is blocked by some atypical antipsychotic drugs (AAPDs), and is considered a model of psychosis [17, 20]. AAPDs are more effective than typical APDs in attenuating indefinite cognitive deficits in rodents administered PCP for 3–24 days [10, 14, 19, 21, 22] Acute treatment with AAPDs produced transient reversal of scPCP-induced impairment in cognitive measures in rodents. The AAPD, lurasidone (Lur), significantly prevented scPCP-induced novel object recognition (NOR) task deficit in female rats for 3 weeks, after which the deficit in NOR re-emerged. The preventative effect of Lur was blocked by WAY100635, a selective 5-HT_1A_R antagonist [23].

TPA-023 (7-(1,1-dimethylethyl)-6-(2-ethyl-2H-1,2,4-triazol-3-ylmethoxy)-3-(2-fluorophenyl)-1,2,4-triazolo[4,3-b]pyridazine), also known as MK-0777 [24], is a GABA_A_, positive allosteric modulator at the benzodiazapine site with a high affinity for GABA_A_Rs, more specifically, the α_2_GABA_A_ subunit, and may target PV-positive neurons [6, 25, 26]. TPA-023 reversed ketamine-induced impairments in spatial working memory in rhesus monkeys [27]. Positive modulators of extrasynaptic GABA_A_ receptors (e.g. gaboxadol) reverse scPCP-induced NOR deficits [28]. However, negative modulation with the inhibitory neurosteroid, pregnenolone sulfate (PregS), also rescued the deficit in NOR in scPCP-treated mice. A double-blind, placebo-controlled trial of MK-0777 in schizophrenia patients on stable doses of antipsychotic medications showed a trend for improvement in three working memory tasks [6]. However, another study failed to replicate these results [29]. Clinically, TPA-023 caused no detectable memory impairment in a study in healthy volunteers [30].

The aims of the present study were to determine whether TPA-023 alone, or as an adjunct to Lur, could prevent scPCP-induced deficits in NOR, ameliorate the scPCP deficits in NOR and operant reversal learning (ORL), a measure of executive function, acutely, or for a prolonged period, diminish PCP-induced LMA, and whether it affected basal, Lur-, and PCP-induced neurotransmitter efflux in medial prefrontal cortex (mPFC) and dorsal striatum (dSTR) in awake freely moving mice. Our results indicate that TPA-023 alone and in combination with Lur had beneficial effects on cognition and significant effects on neurotransmitter release in both brain regions.

## Materials and methods

### Animals

Experiments were performed in 21/2–3-month-old male C57BL/6J mice (Jackson, MA, USA). In experiment 1, 40 mice (*n* = 10/group) were used for acute dose detection study. In experiment 2, for potentiation studies, a total of 50 mice (*n* = 10/group) were used. In experiments 3 and 4, for prevention and prolonged reversal studies, a total of 60 mice (*n* = 10/group) were used. In experiment 5, for RL study, a total of 50 mice (*n* = 10/group) were used. In experiment 6, for LMA, a total of 40 animals (*n* = 8/group) were used. Each group in the microdialysis experiments consisted of eight mice. We also conducted baseline studies for the effect of TPA-023 and lurasidone (Lur) in normal mice on NOR, RL, and LMA tasks. Neither TPA-023 nor lurasidone disrupted the normal behavioral functioning in all these tasks. For ORL task, 1 week prior to behavioral training, the animals were food deprived to 85–90% of their ad libitum weight with unrestricted access to water in their home cages. All experiments were conducted during the light phase. Food and water were available ad libitum. All experiments were conducted in accordance with Institutional Animal Care and Use Committee of Northwestern University, Chicago, IL.

#### Drugs

TPA-023 was purchased from Tocris Bioscience (Ellisville, MO). Lurasidone was supplied by Sumitomo Dainippon Pharmaceuticals, Osaka, Japan. PCP was supplied as a generous gift from the National Institute of Drug Abuse (Bethesda, MD). Lurasidone was suspended in 0.5% methylcellulose and 0.2% Tween80. TPA-023 and PCP were dissolved in saline solution (0.9% NaCl). All drugs were administered intraperitoneally (i.p.) at a volume of 10 ml/kg body weight.

#### Drug Treatment(s)

In experiments 1a–f, 2a–f, 3 and 4, mice were randomly assigned to vehicle- and PCP-treated groups. The vehicle-treated animals received saline (0.9% NaCl) or (0.5% methylcellulose and 0.2% Tween80) and the PCP treatment group received PCP (10 mg/kg) twice a day for 7 consecutive days (days 1–7). They were then subjected to a 7-day washout period (days 8–14) prior to NOR testing [31, 32]. In the prevention study (experiment 2a–c), animals were randomly assigned to vehicle, PCP, or TPA-023+PCP groups. Vehicle controls received saline (0.9% NaCl) and the PCP treatment group received PCP (10 mg/kg) twice a day for 7 days, followed by a 7-day washout period (days 8–14) prior to NOR testing. Mice in the TPA-023+PCP group were given sc TPA-023 (2 mg/kg; b.i.d. 7 days) 30 min prior to each injection of PCP on days 1–7 of the scPCP regimen. We have studied the effect of scPCP (10 mg/kg) in mice and found that scPCP induced robust NOR deficits for upto 9 weeks (Rajagopal et al., unpublished). In experiment 2d–f, for prolonged reversal study, animals were randomly assigned to vehicle, PCP, or PCP+TPA-023 groups. Vehicle controls received saline (0.9% NaCl) and the PCP treatment group received PCP (10 mg/kg; i.p.) twice a day for 7 days, followed by a 7-day washout period (days 8–14) prior to NOR testing. Mice in the PCP+TPA-023 group were given sc TPA-023 (2 mg/kg; b.i.d., 7 days) on days 15–21 following scPCP treatment and washout (days 1–7 treatment; days 7–14 washout). In experiment 4, LMA was tested in acute PCP-treated animals. Male C57BL/6J mice were randomly assigned to three groups. Group 1 received acute saline (0.9% NaCl) 30 min prior to testing; group 2 animals received PCP (10 mg/kg) 30 min prior to testing. Group 3 animals received TPA-023 (0.1, 0.3, or 1 mg/kg) 30 min prior to PCP administration and then tested. For experiment 5, for in vivo microdialysis, vehicle, PCP, or TPA-023 were administered (i.p.) in a volume of 0.1 ml/10 g body weight to randomly assigned animals (*n* = 8 per group). Schema for drug treatments, methods and statistical analyzes: please see supplementary sections SM1a-d, e.

## Results

### Acute administration of TPA-023 dose-dependently ameliorated scPCP-induced deficit in NOR

**Fig. 1:**
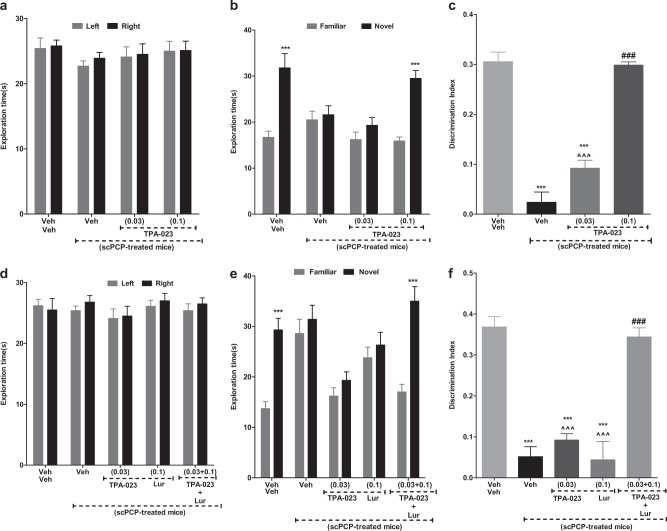
Acute treatment with TPA-023, alone, and in combination with atypical APD, Lurasidone, rescued scPCP-induced NOR deficit in male C57BL/6J mice. **a**–**c** The effect of scVeh, subchronic phencyclidine (scPCP), and scPCP + TPA-023 (0.03 and 0.1 mg/kg) on AT, RT, and DI in scPCP-treated male C57BL/6J mice. Data are shown as mean ± S.E.M. of exploration time (s) *n* = 10 mice per group. **a** AT—the effect of acute administration of TPA-023 (0.03 and 0.1 mg/kg; i.p.) post-scPCP (10 mg/kg i.p. bid for 7 days followed by a 7-day drug-free period) on the exploration of a left and a right object in the 10-min AT in an NOR task in scPCP-treated male C57BL/6J mice; no significant effect in any of the groups tested. **b** RT—the effect of acute TPA-023 (0.03 and 0.1 mg/kg) post-scPCP (10 mg/kg) on the exploration of a novel and a familiar object in the 10-min RT in an NOR task in scPCP-treated male C57BL/6J mice; ***P < 0.001—significant increase in the time spent exploring the novel vs familiar object, Bonferroni *t* test. **c** DI—***P < 0.001—significant reduction in DI vs scVeh group; ^###^*P* < 0.001—significant increase in DI vs scPCP group; ^^^*P* < 0.001—significant decrease in DI vs scPCP+TPA-023 (0.1 mg/kg; i.p.) group. **d**–**f** The effect of scVeh, scPCP, and scPCP + TPA-023 (0.03mg/kg; i.p.), scPCP+Lur (0.1 mg/kg; i.p.), and scPCP+TPA-023 (0.03 mg/kg; i.p.) + Lur (0.1 mg/kg; i.p.) on AT, RT, and DI in scPCP-treated male C57BL/6J mice. Data are shown as mean ± S.E.M. of exploration time (s) *n* = 10 mice per group. **a** AT—no significant difference in the exploration of left and right object between the groups; **b** RT—***P < 0.001—significant exploration of novel vs familiar object; **c** DI—***P < 0.001—significant reduction in DI vs vehicle-treated controls; ^###^*P* < 0.001—significant increase in DI vs scPCP-treated C57BL/6J mice; ^^^*P* < 0.001—significant decrease in DI vs scPCP+TPA-023 (0.03 mg/kg)+Lur(0.1 mg/kg) group

An overall two-way analysis of variance (ANOVA) revealed that neither scPCP nor acute treatment with TPA-023 in either the vehicle or scPCP-treated mice produced any significant effect on object exploration time during acquisition trial (AT), [*F*_(3,36)_ = 0.411, *P* = 0.96; Fig. 1a]. A two-way ANOVA revealed significant interactions between drug treatment and object exploration times in the retention trial (RT), [*F*_(3,36)_ = 33.97; **P < 0.01; Fig. 1b]. Post-hoc analysis revealed that saline- but not the scPCP-treated group explored the novel object significantly more than the familiar object (***P < 0.001; Fig. 1b). Acute TPA-023, 0.1 but not 0.03 mg/kg, reversed the scPCP-induced NOR deficit (***P < 0.001; Fig. 1b). One-way ANOVA analysis of the discrimination index (DI) showed significant differences between the groups, [*F*_(3,36)_ = 76.63;***P < 0.001; Fig. 1c]. The DI for scPCP-treated mice was significantly reduced (***P < 0.001) vs saline-treated control mice. The DI for the scPCP-treated animals given TPA-023 (0.1, but not 0.03 mg/kg) significantly increased the DI vs scPCP+veh group (*P* < 0.001; Fig. 1c) and was not significantly different from that of the saline-treated control group (*P* = 0.13; Fig. 1c). No significant effect was observed in the total exploration times between the groups.

### Co-administration of sub-effective doses (SEDs) of TPA-023 and lurasidone significantly ameliorated scPCP-induced NOR deficit

An overall two-way ANOVA revealed that none of the treatments had any significant effect on object exploration during AT [*F*_(4,45)_ = 1.004, *P* = 0.91; Fig. 1d]. In RT, a two-way ANOVA revealed significant interactions between drug treatments and object exploration times [*F*_(4,45)_ = 27.60; ***P < 0.001; Fig. 1e]. Post hoc analysis revealed that the vehicle-treated control animals had a clear preference for novel vs familiar object (***P < 0.001; Fig. 1e). This effect was absent in the mice treated with scPCP. The ScPCP-treated mice given lurasidone (0.1 mg/kg) or TPA-023 (0.03 mg/kg; see Fig. 1e) did not ameliorate scPCP-induced NOR deficit, indicating that these are SEDs of both drugs. However, co-administration of SED TPA-023 (0.03 mg/kg) + SED lurasidone (0.1 mg/kg) to scPCP-treated animals significantly reversed scPCP-induced NOR deficit (***P < 0.001; Fig. 1e). A one-way ANOVA analysis of the DI showed significant group differences [*F*_(4,45)_ = 33.19; ***P < 0.001; Fig. 1f). The DI for scPCP-treated mice was significantly reduced (***P < 0.001) vs vehicle-treated mice. The DI for the scPCP-treated animals co-administered SED TPA-023 + SED lurasidone, was significantly increased vs scPCP+veh group (^###^*P* < 0.001; Fig. 1f) and not significantly different from that of vehicle-treated mice (*P* = 0.10). No significant effect was observed in the total exploration times between the groups.

### Pretreatment with TPA-023 prevented scPCP-induced NOR deficit but only for 5 weeks

**Fig. 2:**
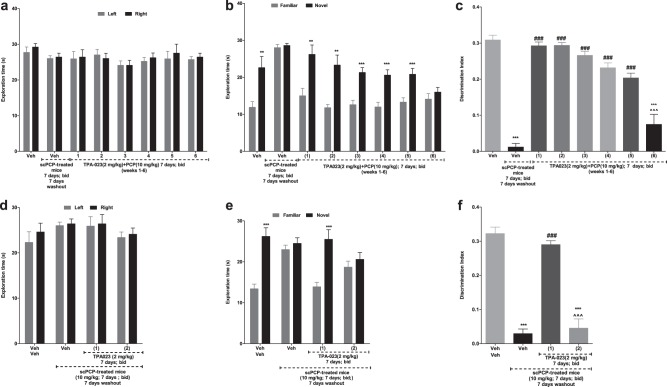
Subchronic TPA-023 treatment prevented and showed prolonged rescue of scPCP-induced NOR deficit. **a**–**c** The effect of vehicle, scPCP, and scTPA-023 (2 mg/kg; i.p.; 7 days; days 0–7) + scPCP (10 mg/kg; i.p.; b.i.d.; 7 days; days 0–7) 30 min after scTPA-023 injections on NOR on AT, RT, and DI in scPCP-treated male C57BL/6J mice. Data are shown as mean ± S.E.M. of exploration time (s) *n* = 10 mice per group. For the scPCP and scVeh groups, the average for all the weeks they were tested, calculated, and reported as a single histogram bar. **a** AT—no significant difference in the exploration of left and right object between the groups; **b** RT—**P < 0.01; ***P < 0.001—significant exploration of novel vs familiar object; **c** DI—***p < 0.001—significant reduction in DI vs vehicle-treated controls; ^###^*P* < 0.001—significant increase in DI vs scPCP-treated C57BL/6J mice; ^^^*P* < 0.001: significant decrease in DI vs scTPA-023+scPCP week 6. **d**–**f** The effect of vehicle, scPCP, and scPCP (10 mg/kg; i.p.; b.i.d.; 7 days; days 0–7; followed by 7 days washout) + scTPA-023 (2 mg/kg; i.p.; 7 days; days 15–21) on NOR on AT, RT, and DI in scPCP-treated male C57BL/6J mice. Data are shown as mean ± S.E.M. of exploration time (s) *n* = 10 mice per group. For the scPCP and scVeh groups, the average for all the weeks they were tested, calculated, and reported as a single histogram bar. **a** AT—no significant difference in the exploration of left and right object between the groups; **b** RT—***p < 0.001—significant exploration of novel vs familiar object; **c** DI—***p < 0.001—significant reduction in DI vs vehicle-treated controls; ^###^*P* < 0.001—significant increase in DI vs scPCP-treated C57BL/6J mice; ^^^*P* < 0.001: significant decrease in DI vs scPCP+scTPA-023 (week 1)

An overall repeated-measures ANOVA did not show any significant effect on object exploration in the AT of any group [*F*_(7,72)_ = 1.01, *P* = 0.93; Fig. 2a]. There was significant interaction between drug treatment and object exploration times in RT [*F*_(7,72)_ = 21.13;***P < 0.001; Fig. 2b]. Post-hoc analysis revealed that saline, but not the scPCP-treated mice, explored novel significantly more vs familiar object (**P < 0.01). For the vehicle and scPCP groups, the 6-week averages are shown as a single histogram bar (Fig. 2b). Pretreatment with scTPA-023 (2 mg/kg) followed by scPCP (10 mg/kg) significantly prevented the onset of NOR deficits for up to 5 weeks (***P < 0.001; ***P < 0.001; ***P < 0.001; **P < 0.01; *P < 0.05 weeks 1–6; Fig. 2b). The DI showed significant interactions between groups [*F*_(7,72)_ = 56.15, ***P < 0.001; Fig. 2c]. The DI for scPCP-treated mice was significantly reduced vs control mice (***P < 0.001; Fig. 2c). The DI for mice treated with TPA-023 prior to each injection of PCP was the same as that for control mice at each week for up to 5 weeks (^###^*P* < 0.001; Fig. 2c). No significant effect was observed in the total exploration times between the groups.

### Sc TPA-023 administration following scPCP treatment reversed scPCP-induced NOR deficit but only for 1 week

An overall repeated-measures ANOVA did not show any significant effect on object exploration in the AT of any of the groups [*F*_(3,36)_ = 0.56, *P* = 0.65; Fig. 2d]. In RT, there was a significant interaction between drug treatment and object exploration times [*F*_(3,36)_ = 28.39; ***p < 0.001; Fig. 2e]. Further post hoc Bonferroni analysis revealed that saline, but not the scPCP-treated group, explored novel significantly more vs familiar object (***P < 0.001). The average for all weeks were calculated and shown as a single histogram bar. ScTPA-023 (2 mg/kg) in scPCP (10 mg/kg)-treated animals showed a significant reversal of PCP-induced deficits for only one 1 week (***P < 0.001; Fig. 2e). In the DI, there was significant interaction between groups [*F*_(3,36)_ = 18.53, ***P < 0.001; Fig. 2f]. The DI for scPCP-treated mice given saline was significantly reduced (***P < 0.001; Fig. 2f). The DI for mice treated with scTPA-023 following scPCP was significantly increased to normal levels 1 week after withdrawal vs the scPCP mice (^###^*P* < 0.001; Fig. 2f); however, the DI in these mice was not significantly different from that of the scPCP-treated mice by week 2, indicating that a pathological process leading to a deficit in NOR had now emerged. No significant differences were observed in the total exploration times between any of the groups.

### Acute TPA-023 treatment significantly reversed scPCP-induced RL deficit

**Fig. 3:**
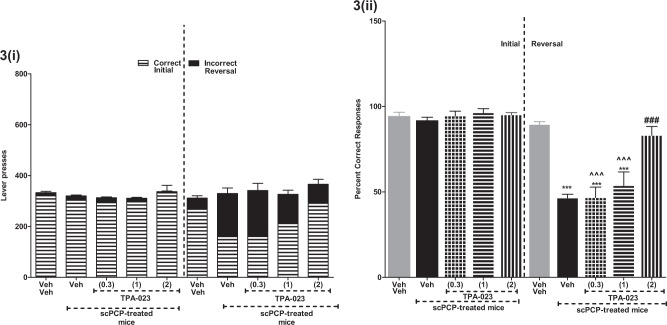
Acute TPA-023 treatment significantly reversed scPCP-induced reversal learning deficit. i, ii The effect of acute treatment with TPA-023 (0.3, 1, and 2 mg/kg) post-scPCP (10 mg/kg; 7 days; b.i.d.) administrations on the performance of the initial and reversal phase of the reversal learning task (n = 10/group). i Correct/incorrect lever presses—Data are shown as the mean total number of lever presses. Correct responses are shown as bars shaded as cross lines, and incorrect responses are shown as filled bars in black. ii Percent correct responses—***P < 0.001—significant reduction in percent correct responses vs scVeh; ^###^*P* < 0.001—significant increase in percent correct responses vs scPCP; ^^^*P* < 0.001—significant reduction in percent correct responses vs scPCP+TPA-023 (2 mg/kg)

There was no significant effect on total lever pressing in the initial or reversal phases on any of the groups tested, suggesting that LMA was not affected. There was a significant reduction in percent correct responses (PCRs) in the reversal phase vs the initial phase in the scPCP-treated group (***P < 0.001; paired *t* test; Fig. 3(ii). A one-way ANOVA on the reversal phase showed a significant effect of scPCP treatment on PCRs [*F*_(4,45)_ = 23.93; ***P < 0.001; Fig. 3(ii)]. The post hoc Bonferroni analysis revealed that TPA-023, 2, but not 0.3 or 1 mg/kg, attenuated scPCP-induced deficit in PCRs (^###^*P* < 0.001; Fig. 3(ii); reversal), i.e., rescued scPCP-induced RL deficit. Auxiliary measures: See ST1 for results and statistical analyzes. Conclusion: These data indicate that TPA-023 dose dependently rescued scPCP-induced RL deficit; and that the SED is 1 mg/kg and the ED is 2 mg/kg.

### Acute TPA-023 dose-dependently blocked PCP-induced LMA

**Fig. 4:**
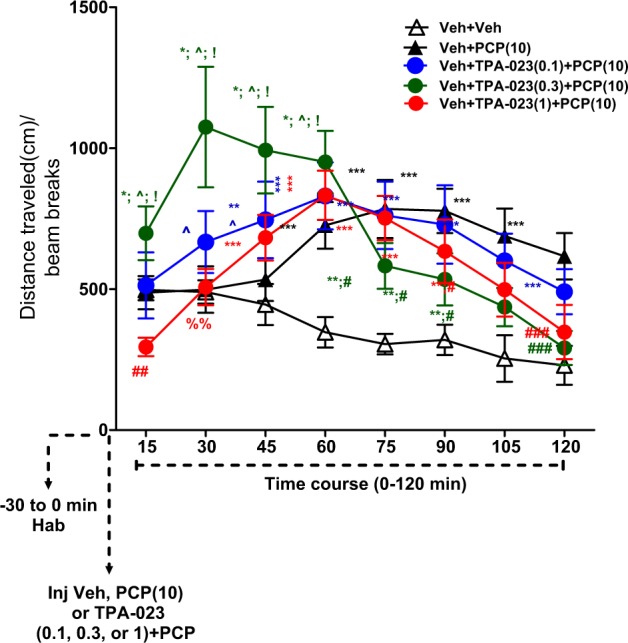
Acute TPA-023 showed trends to block PCP-induced locomotor activity at different time points at different doses in normal mice. Time course effect for the distance traveled (cm) effect for Veh+veh, Veh + PCP (10 mg/kg); Veh + TPA-023 (0.1, 0.3, or 1 mg/kg) + PCP (10 mg/kg) on LMA in male C57BL/6J mice. Data are presented as group means ± SEM for eight successive 15-min intervals. *P < 0.05; **P < 0.01; ***P < 0.001: significant increase in LMA vs Veh+veh; ^*P* < 0.05: significant increase in LMA vs Veh+PCP (10); ^#^*P* < 0.05; ^###^*p* < 0.001: significant decrease in LMA vs Veh+PCP (10); ^%%^*P* < 0.01: significant decrease in LMA vs Veh+TPA-023 (0.3)+PCP (10); ^!^*P* < 0.05: significant increase in LMA vs Veh+TPA-023 (1)+PCP (10)

A repeated-measures ANOVA conducted on the time course effect showed significant interactions between the treatment groups×distance traveled (cm) [*F*_(4,35)_ = 7.41; **P < 0.01; Fig. 4). The Veh+PCP (10 mg/kg) group showed a significant increase in the LMA vs Veh+Veh group from 60 to 120 min (***P < 0.001; Fig. 4). The Veh+TPA-023 (0.1 mg/kg) + PCP (10 mg/kg) group did not block PCP-induced increase in LMA (15–120 min; Fig. 4). The Veh+TPA-023 (0.3 mg/kg) + PCP (10 mg/kg) group showed partial blockade of PCP-induced increase in LMA during 60–105 min and showed complete blockade during the 105–120 min time point (^###^*P* < 0.001; Fig. 4). The Veh+TPA-023 (1 mg/kg) + PCP (10 mg/kg) group significantly showed reduction in LMA vs Veh+PCP during 0–30 min time points (^##^*P* < 0.01; Fig. 4). Although this group was not different from the Veh+PCP(10 mg/kg) group during the 30–90 min time points, there was partial blockade during the 90–105 min time period and complete blockade of PCP-induced increase in LMA during the 105–120 min time period (^###^*P* < 0.001; Fig. 4). This study showed that TPA-023 has strong trends to block PCP-induced hyperactivity, indicating potential efficacy as a treatment for psychosis.

### Effect of TPA-023 on basal and lurasidone-induced neurotransmitter efflux (NT) in mPFC and dSTR

**Fig. 5:**
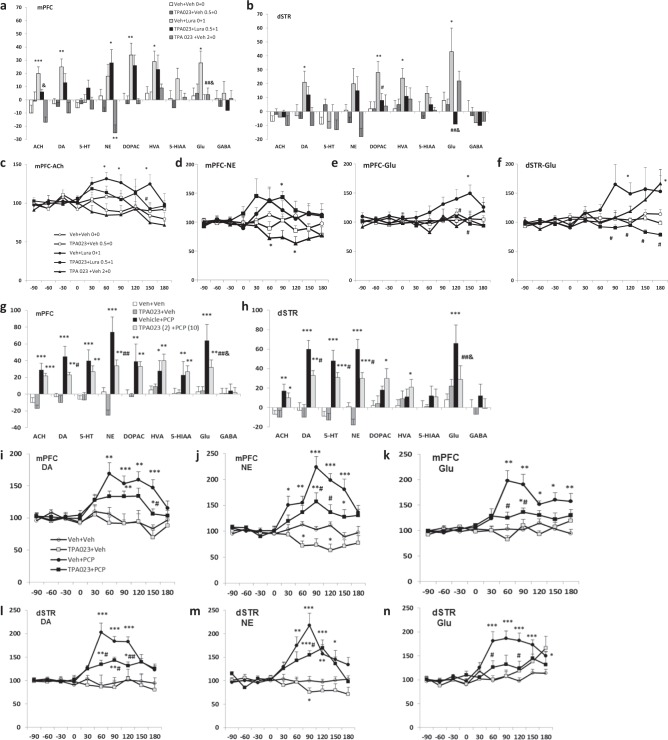
Effect of TPA-023 on lurasidone and PCP-induced neurotransmitter efflux in mPFC and dSTR. **a**–**f** Effect of TPA-023 on basal and lurasidone-induced NT efflux in mPFC and dSTR. **a**, **b** AUC values (*y* axis) for all the NTs (*x* axis), and **c**–**f** time–response curves, with *x* axis presenting time points (first injection was at −30 time points, and second injection was at 0 time points), and *y* axis presenting percentage of baseline. TPA-023, at dose of 0.5 and 2.0 mg/kg, mg/kg, had no significant effects on NT efflux in microdialysate in either mPFC (**a**) or dSTR (**b**). The high-dose TPA-023 decreased NE efflux in mPFC (**b**, **d**). Lurasidone, at dose of 1.0 mg/kg, significantly increased ACh, DA, NE, DOPAC, HVA, and Glu in mPFC (**a**) and DA, DOPAC, HVA, and Glu efflux in dSTR (**b**). TPA 023, 0.5 mg/kg, given 30 min prior to lurasidone, significantly decreased lurasidone-induced mPFC increase in Glu (**a**, **e**) and dSTR DOPAC and Glu (**b**, **f**). After pretreatment with TPA-023, lurasidone failed to increase any NTs in either regions when compared to vehicle group. *P < 0.05, **P < 0.01, ***P < 0.001 vs Veh+Veh group, ^#^*P* < 0.05, ^##^*P* < 0.01 vs Veh+Lura group, and ^&^*P* < 0.05 drug interaction (TPA-023×Lurasidone). **g**–**n** Effect of TPA-023 on acute PCP-induced NT efflux in mPFC and dSTR. **g**, **h** AUC values (*y* axis) for all the NTs (*x* axis), and **c**–**h** time–response curves, with *x* axis presents time points (first injection was at −30 time points, and second injection was at 0 time points), and *y* axis presents percentage of baseline. In mPFC (**g**), PCP (10 mg/kg, i.p.) significantly increased ACh, DA, 5-HT, NE, and Glu efflux in both mPFC (**g**) and dSTR (**h**). Given 30 min prior to PCP injection, TPA-023 (2.0 mg/kg) significantly suppressed PCP-induced DA (**i**), NE (**j**), and Glu (**k**) efflux in mPFC and DA (**i**), 5-HT, NE (**m**), and Glu (**n**) efflux in dSTR. *P < 0.05, **P < 0.01, ***P < 0.001 vs Veh+Veh group, ^#^*P* < 0.05, ^##^*P* < 0.01 vs Veh+PCP group, and ^&^*P* < 0.05 drug interaction (TPA-023×PCP)

Systemic administration of TPA-023, at 0.5 and 2.0 mg/kg, had no significant effects on NT efflux in microdialysates in either mPFC (Fig. 5a) or dSTR (Fig. 5b), except that the higher dose of TPA-023 slightly but significantly decreased NE efflux in mPFC (**P = 0.005, vs vehicle, Fig. 5b, d). Lur, 1.0 mg/kg, significantly increased acetylcholine (ACh) (***P < 0.001), dopamine (DA) (**P = 0.001), norepinephrine (NE) (*P = 0.030), dihydroxyphenylacetic acid (DOPAC) (**P = 0.003), homovanillic acid (HVA) (*P = 0.032), and Glu (*P = 0.014) in mPFC (Fig. 5a) and DA (*P = 0.020), DOPAC (**P = 0.004), HVA (*P = 0.019) and Glu (*P = 0.038) efflux in dSTR (Fig. 5b). TPA-023, 0.5 mg/kg, given 30 min prior to Lur, 1.0 mg/kg, significantly decreased the Lur-induced mPFC Glu (^##^*P* = 0.007, vs Lur alone, Fig. 5a, e), as well as the dSTR DOPAC (^#^*P* = 0.027) and Glu (^##^*P* = 0.003, Fig. 5b, f) efflux. After pretreatment with TPA-023, DA efflux from Lur was non-significantly reduced in both regions vs vehicle group. A two-way ANOVA showed a significant interaction between pretreatment (vehicle, TPA-023) and treatment (Lur) on the efflux of cortical ACh (*F*_(3,30)_ = 4.761, ^&^*P* = 0.037, drug interaction, Fig. 5a, c), Glu (*F*_(3,30)_ = 4.678, ^&^*P* = 0.039, Fig. 5a, e), and dSTR Glu (*F*_(3,30)_ = 4.434, ^&^*P* = 0.044, Fig. 5b, f).

### Effect of TPA-023 on PCP-induced NT efflux in mPFC and dSTR

In mPFC (Fig. 5g), PCP (10 mg/kg, i.p.) increased ACh (***P < 0.001 vs vehicle), DA (***P < 0.001), 5-HT (***P < 0.001), NE (***P < 0.001), DOPAC (**P = 0.001), HVA (*P = 0.015), 5-HIAA (**P = 0.009), and Glu efflux (***P < 0.001). PCP also increased dSTR (Fig. 5h) ACh (**P = 0.001), DA (***P < 0.001), 5-HT (***P < 0.001), NE (***P < 0.001), and Glu (*P* < 0.001) efflux. Given 30 min prior to PCP injection, TPA-023 (2.0 mg/kg) significantly suppressed PCP-induced DA (^#^*P* = 0.012, vs PCP, Fig. 5i), NE (^##^*P* = 0.002, Fig. 5j), and Glu (^##^*P* = 0.004, Fig. 5k) efflux in mPFC, and DA (^#^*P* = 0.032, Fig. 5l), 5-HT (^#^*P* = 0.020), NE (*P* = 0.015, Fig. 5m), and Glu (^##^*P* = 0.009, Fig. 5n) efflux in dSTR. Two-way ANOVA showed significant interaction between pretreatment (TPA-023) and treatment (PCP) on the efflux of cortical Glu (*F*_(3,28)_ = 5.439, ^&^*P* = 0.027) and dSTR Glu (*F*_(3,27)_ = 7.252, ^&^*P* = 0.012).

## Discussion

The major findings from this study in male C57BL/6J mice were: (1) acute treatment with TPA-023 transiently restored episodic memory and executive functioning deficits in scPCP-treated mice; (2) TPA-023 treatment administered prior to each of the 14 injections of PCP significantly prevented scPCP-induced NOR deficit for up to 5 weeks and showed prolonged reversal of scPCP-induced NOR deficit for 1 week; (3) co-administration of SED TPA-023 and SED lurasidone acutely restored NOR in scPCP-treated mice; (4) TPA-023 at two doses and two different time points diminished PCP-induced increase in LMA between 45 and 120 min time intervals; (5) TPA-023 inhibited the increases in cortical ACh, DA, and glutamate as well as the dSTR increases in DA and Glu produced by lurasidone; and (6) TPA-023 inhibited PCP-induced cortical and striatal DA, 5-HT, NE, and Glu efflux.

### Acute rescue of NOR by TPA-023

NOR task is dependent upon synaptic plasticity, which includes long-term potentiation (LTP) and successful integrated activity of a variety of GABAergic interneurons and principal neurons in the HIP, PFC, and other brain regions [33, 34]. We observed significant rescue of scPCP-induced LTP impairment by acute TPA-023 treatment (unpublished). Acute treatment with TPA-023 was effective to rescue NOR in scPCP-treated mice. However, this lasted <24 h. The extent of the recovery and the time course matched with that of a single dose of a variety of AAPDs and contrast with the negative effects of haloperidol in this model. The effect of TPA-023 is also identical to the combined effect of 5-HT_2A_R inverse agonism, 5-HT_1A_R partial agonism, and a weak D_2_R blockade [10, 19, 35, 36]. The rapidity with which TPA-023 and lurasidone restore NOR suggests that all the molecular components needed for the synaptic changes crucial for learning and memory are present in the scPCP-treated mice and that neither new protein synthesis nor neurogenesis is needed. Thus the effects of the PCP regimen are to disrupt the cascades of molecular events that are required for LTP and other cognitive mechanisms and perhaps what TPA-023 and AAPDs do is to inhibit or remove the molecular changes that cause PCP to disrupt NOR when given acutely and are present in some form after washout of PCP following scPCP treatment. This effect of TPA-023 is consistent with a previous report that gaboxadol, a GABA_A_R agonist, restores NOR in scPCP-treated rats [28]. We have also found that gaboxadol rescues NOR in scPCP-treated male C57BL/6J mice (Rajagopal et al. unpublished data). PregS, an endogenous neurosteroid, which negatively modulates GABA_A_Rs, has a similar effect suggesting that some specific components of GABA_A_R signaling are involved in each case.

### Combination of SED TPA-023 and Lur rescues NOR

SED of TPA-023 (0.03 mg/kg) in combination with SED lurasidone (0.1 mg/kg) reversed scPCP-induced NOR deficits. We used lurasidone (Fig. 2) as this AAPD, with multi-receptor mechanism, has shown significant efficacy in several domains of cognition, negative symptoms, as well as positive symptoms without any undue side effect profiles in our preclinical studies ([23]. Our initial goal is to investigate the transient effect of interactions between TPA-023 and lurasidone. The long-term effects of the combination treatment of TPA-023 and Lur in the future is indicated.

This suggests that TPA-023 may be a valuable adjunctive treatment for CIAS in combination with Lur or other AAPDs with similar pharmacology, e.g., olanzapine and risperidone. The effect of Lur itself to rescue NOR in the scPCP-treated mice is blocked by pretreatment with the GABA_A_R antagonist, gabazine, which indicates that stimulation of GABA_A_R by gaboxadol is required for the transient rescue of Lur (Meltzer et al., unpublished). This supports the conclusion that the efficacy of the combination of PregS and Lur to rescue NOR is based on stimulation of GABA_A_Rs. Whether TPA-023 would be effective to treat CIAS in patients receiving effective doses of AAPDs for treatment of psychosis, or perhaps only at SEDs, remains to be determined.

### Prevention of scPCP-induced deficit in NOR by combined pretreatment with scTPA-023

Administration of Lur prior to each injection of PCP has previously been shown to block the effect of PCP to produce a persistent deficit in NOR, but for only 14 days, with re-emergence of the NOR deficit between day 14 and day 21 [23, 36]. TPA-023 was also able to prevent scPCP from impairing NOR that persisted 5 weeks, with the deficit in NOR partially present at week 6, indicating a step-wise decrease in the rescue mechanism. The length of the prevention of the deficit in NOR was 3 weeks longer than that of Lur alone, indicating that TPA-023 may have particular benefit for prevention of adverse effects of PCP. However, higher doses of Lur need to be studied to affirm this conclusion. When used in a regimen comparable to that of Lur, risperidone (0.5 mg/kg, i.p.), another AAPD, did not show a protective effect for the prevention of the deficit in NOR [37]. The prevention of the PCP-induced deficit for a prolonged period suggests that GABA_A_R agonism may be able to prevent cumulative effects of PCP, perhaps on gene expression, that interfere with learning and memory. These effects persist long after both PCP and TPA-023 are no longer present in the brain. It is conceivable that neurogenesis or restoration of connectivity between different brain areas contributes to this recovery but that without additional treatment with TPA-023 these processes cease to occur or be effective. We tested higher doses in order to have higher concentrations of TPA-023 present throughout the period in which PCP might produce enduring damage to the process required for learning and memory. It is of particular interest that treatment of rats with PCP, 2.5 mg/kg daily for 5 days, reduced cortical PV mRNA levels and expression of PV mRNA and a subunit of the Kv3.1 voltage-gated potassium channel, which is believed to confer fast spiking properties on PV interneurons [38]. These changes were reversed by chronic clozapine treatment (time not specified). The authors suggest that PCP may have caused cell death but that seems unlikely in light of the ability of clozapine in that study or Lur or TPA-023, in our studies, to reverse PCP-induced NOR deficit. Interestingly, we have found the most robust genetic predictor of improvement in psychopathology by Lur in acutely psychotic schizophrenic patients was a polymorphism in the potassium two-pore channel gene, KCNK9 (Li et al., in press).

### Persistent rescue of the scPCP-induced deficit in NOR by post-PCP administration of TPA-023

TPA-023 given daily for 7 days to scPCP mice with a persistent deficit in NOR was able to produce a prolonged rescue of NOR but only for 1 week. This contrasts with the brief rescue (24 h) produced by a single dose of TPA-023 (Fig. 1a–c) and the prolonged prevention of the deficit in NOR produced by TPA-023 when given with PCP. During the time when TPA-023 restored NOR, the synaptic events needed for encoding, consolidation, and retrieval of novel object memory were able to function at least equal to that of the normal mice (Huang et al., unpublished). However, the normal mice and mice given TPA-023 may not have performed these activities in the same manner. We have measured neurotransmitter release during NOR in the TPA-023-rescued, Lur-rescued, and normal mice and noted significant differences in the release of DA, ACh, Glu, and GABA in the HIP and PFC regions in these groups (Huang et al, unpublished).

Novel mechanisms that enable NOR may be the consequence of these post-PCP treatments. The return of the NOR deficit in the TPA-023 mice at the end of the second week suggests that PCP-initiated disruptive genomic processes were suppressed but not totally eliminated by scTPA-023 treatment (Li et al., unpublished). Identification of those re-emerging abnormalities in synaptic plasticity could provide information about the development of CIAS and the mechanism of action of TPA-023 and AAPDs and future studies is indicated.

PCP and MK-801, both non-competitive NMDAR antagonists, prevent the induction of LTP in the dentate gyrus and hippocampal CA1 regions [34, 39, 40]. Loss of PV interneurons or markers thereof has been reported in scPCP-treated rodents [37, 38, 41–43] and in individuals with schizophrenia [44].  Other possibilities for the disruption of cognition in the NMDAR antagonists-treated rodents other than abnormalities in parvalbumin-positive interneurons should be considered, including, but not restricted to elevated density of cFOS positive interneurons in the hippocampus after repeated NMDAR antagonist treatment [45]. Most recently, we observed that the excitatory synaptic transmission in scPCP-treated animals in CA1 pyramidal neurons needed for LTP induction was more strongly inhibited by excessive GABAergic activity vs control mice, as indicated by significant enhancement in the amplitude of GABA inhibitory postsynaptic currents in hippocampal slices from scPCP-treated mice vs controls [34]. We also observed a strong trend of reversal of scPCP-induced LTP impairment in the scPCP mice given acute TPA-023 (unpublished). These results suggest a novel and previously unreported elevation in GABA signaling in the HIP that could contribute to the cognitive dysfunction in NMDAR antagonist-treated rodents. Because TPA-023 is a subtype-selective partial agonist at α_2,3_ and an α_1/5_ antagonist, further study is needed to determine which of these effects, or perhaps other unknown but related actions, is responsible for its effects in the scPCP-treated mice.

### Effect of TPA-023 on LMA

Acute administration of PCP to rodents increases LMA, stereotypy, and ataxia [46]. The increase in LMA and stereotypy are thought to mimic psychosis in schizophrenia [47]. By itself, at the doses tested, TPA-023, 0.3 mg/kg, enhanced the LMA produced by PCP 10 mg/kg for the first 60 min after administration, while 0.1 mg/kg showed enhanced LMA between 15 and 45 min. However, 1.0 mg/kg significantly reduced LMA during the initial 30 min, after which it was comparable to the PCP group (with no enhanced LMA). Pretreatment with TPA-023, 0.3 and 1 mg/kg, produced partial and complete inhibition of PCP- induced LMA between 90 and 120 min (Fig. 4). The increased LMA produced by PCP has been suggested to be the result of 5-HT or DA release, or both, in the striatum [20]. Acute administration of PCP, 10 mg/kg, to mice significantly increases efflux of cortical and dSTR ACh, DA, 5-HT, NE, and Glu (Fig. 5; [48]. These increases are partially inhibited by the GABA_A_R agonist gaboxadol and TPA-023 (Meltzer and Huang, unpublished). As will be discussed, TPA-023 alone significantly inhibited PCP-induced neurotransmitter efflux.

### Effect of TPA-023 on Lur- and PCP-induced neurotransmitter efflux in mPFC and dSTR

#### Neurotransmitter release and cognitive function

We, and others, have previously suggested that release of DA, ACh, and Glu in mPFC, HIP, or dSTR by AAPDs could contribute to their ability to improve NOR and RL in scPCP-treated mice [49, 50]. TPA-023, by itself, did not alter the release of neurotransmitters in the mPFC, although it might do so during the course of the NOR task. We did not detect any effect of Lur on basal release of DA, NE, ACh, Glu, GABA, or 5-HT in normal or scPCP-treated mice in this study (Huang et al. unpublished data). However, TPA-023, 0.5 mg/kg, 30 min prior to Lur, 1.0 mg/kg, inhibited the release of DA, ACh, and Glu in the mPFC and DA and Glu in dSTR. It had no effect on the efflux of 5-HT or 5-HIAA in either region. TPA-023 completely eliminated the release of Glu in both regions but only partially blocked the release of DA and ACh. This indicates that Glu release in these two regions is not necessary for the recovery of NOR. Some increase in DA release in the mPFC and dSTR and ACh release in the mPFC may be necessary for the recovery of NOR. The doses of TPA-023 and Lur used in microdialysis are close to the doses used in the ORL but much higher than those used in the NOR tests. We did not test the lower dose of TPA-023 in microdialysis since TPA-023, 0.5 mg/kg had no effect on basal NT efflux. At the lower doses used in NOR, it is possible that TPA-023, could induce other effects, e.g., release of neurotrophins such as BDNF or neuregulin, which could, in part, rescue scPCP-induced NOR deficit.

#### Neurotransmitter release and LMA

The ability of acute PCP to stimulate Glu release in mPFC and dSTR could be the basis for its enhancement of LMA [11]. As noted above, TPA-023 suppressed PCP-induced monoamine NT and Glu efflux in both mPFC and dSTR. Acute PCP has been suggested to enhance Glu release, at least in part, by disinhibiting GABA neurotransmission, via blockade of NMDARs on GABA interneurons [51, 52]. TPA-023 administration prior to PCP could inhibit overexcitation of pyramidal neurons by diminishing Glu release. Other AAPDs, including aripiprazole, olanzapine, and clozapine [53, 54], also have been reported to suppress PCP- and MK801-induced cortical Glu and 5-HT efflux, suggesting that modulating cortical 5-HT or Glu release might be a shared property of AAPDs and TPA-023 and a basis for their efficacy as combined treatment for CIAS. In our findings, the NT efflux is partially blocked by the GABA_A_R partial agonist, TPA-023, via stimulation of GABAARs, which in turn disinhibited the disinhibition on the glutamatergic principal neurons (induced by PCP treatment). PCP-induced excitatory NT efflux is believed to be in part the cause of hyperactivity as well as the cognitive deficit in lone-term treatments [11, 13]. Thus the ability of TPA-023 to attenuate PCP-induced monoamine and Glu efflux in both regions may explain its effect to reverse PCP-induced hyperactivity and cognitive deficits.

## Summary and conclusions

Acute treatment with TPA-023, a GABA_A_ α_2,3_ subtype-selective partial agonist and α_1/5_ antagonist, restored NOR and ORL in scPCP-treated mice for <2 days. ScTPA-023 also produced a prolonged recovery from the cognitive impairing effects of scPCP treatment but for 1 week only. TPA-023 administration prior to each PCP dose during treatment with PCP for a week also prevented the prolonged deficit in NOR, but the deficit emerged after 5 weeks. Similar actions have previously been reported to be produced by several other AAPDs. Potential clinical use is supported by our finding that an SED of TPA-023 in combination with an SED of the AAPD, Lur, restored NOR in scPCP-treated mice. TPA-023 at higher doses diminished the LMA produced by PCP, suggesting an antipsychotic-like action. The effects of TPA-023 on the effects of Lur and PCP on neurotransmitters, especially Glu and DA indicated possible mechanisms by which TPA-023 produced these effects. These findings suggest that further study of TPA-023 to treat schizophrenia, alone and in combination with AAPDs, is indicated.

## Electronic supplementary material

TPA-023 attenuates subchronic phencyclidine-induced declarative and reversal learning deficits via GABAA receptor agonist mechanism: possible therapeutic target for cognitive deficit in schizophrenia
