# Supplementary material for: TPA-023 attenuates subchronic phencyclidine-induced declarative and reversal learning deficits via GABAA receptor agonist mechanism: possible therapeutic target for cognitive deficit in schizophrenia
Source: Neuropsychopharmacology. 2018 Jul 23;43(12):2468–77. doi: 10.1038/s41386-018-0160-3 (PMC6180114; doi:10.1038/s41386-018-0160-3)
Supplement: Supplementary file 1 — TPA-023 attenuates subchronic phencyclidine-induced declarative and reversal learning deficits via GABAA receptor agonist mechanism: possible therapeutic target for cognitive deficit in schizophrenia [file 41386_2018_160_MOESM1_ESM.docx]

**SM1. Methods**

**SM1a.** **Novel Object Recognition Task**

**SM1a (i) Apparatus -** The NOR testing in mice was adapted from Hashimoto et al (Hashimoto et al, 2005), and was slightly modified (i.e. size of the box, usage of white background to the walls of the box instead of black reflective surfaces, and duration of the trials) based on prelimary experiments (Rajagopal *et al*, 2016a; Rajagopal *et al*, 2014). Prelminary studies established that when black reflective surfaces were used for the inner surfaces of the NOR box, the animals failed to explore the objects. The same was true when larger objects were used. Hence, white walls and small objects for exploration were used throughout. The NOR apparatus consisted of an open box made of Plexiglas (52 cm L; 52 cm W; 31 cm H) with white walls and a solid floor. The box was positioned approximately 30 cm above the floor centered on a table such that the overhead lights could not provide a spatial cue.

**SM1a.(ii) Test -** Following the 7-day washout from subchronic drug or Veh treatment on each of the three days prior to the acquisition trial, mice were habituated for one hour/day for 3 days as a group to the empty NOR arena. During the acquisition trial, the animals were allowed to explore two identical objects (e.g. A1 and A2) for 10 minutes. This was followed by a 24 hour inter-trial interval (ITI), when the animals were returned to the home cage. During the retention trial, the animals were allowed to explore familiar object (e.g. A) from the acquisition trial and a novel object (e.g. B). The location of the novel object in the retention trial was randomly assigned for each test mouse using a pseudorandom schedule. The pseudorandom sequences followed the criteria suggested by Gellerman, 1933, and thus reduced the effects of object and place preference (Gellermann, 1933). Also, to avoid bias or olfactory trails, we used objects in triplicates. Behavior was recorded on video for blind scoring of object exploration. Object exploration was defined as an animal licking, sniffing, or touching the object with the forepaws while sniffing. Exclusion criteria for the NOR test is defined as; “if the animal fails to explore one or both of the objects for less than 5(s) in the acquisition or retention trial, it is excluded from the final data analyzes”. We did not exclude any data in the studies presented in this article, considering all the animals explored >5s. The exploration time (s) of each object in each trial was recorded manually by the use of two stopwatches. The discrimination index (DI) [(time spent exploring the novel object - time spent exploring the familiar object)/total exploration time] was then calculated for retention trials. We also calculated the total exploration times (TETs), i.e. [(time spent exploring the left & right object in the acquisition trial + time spent exploring the novel + familiar object in the retention trial). In some cohorts, mice were tested twice, with a gap of one week to 10 days between tests, to reduce carryover effects. The validity of this animal sparing method has been reported elsewhere (Miyauchi *et al*, 2016). Also, when the animals were re-tested, new objects were used, i.e. the ones that were not used in the prior test. Furthermore, mice showed stable exploration throughout the study period. We would exclude animals if they failed to do so; however, in our studies, we did not observe any significant changes in their object explorations, and hence, we did not exclude any animal.

**SM1a (iii) Data Analysis:** All data are expressed as the mean ± S.E.M. (N = 10 per group). Exploration data (in the acquisition and retention trials) were analyzed by a two-way analysis of variance (ANOVA) followed by the pair-wise comparison when a significant effect was detected by ANOVA. The DI & TET data were analyzed by one-way ANOVA followed by post hoc Bonferroni test when a significant effect was detected by ANOVA.

**SM1b. Operant Reversal Learning task (ORL) -**

**SM1b(i) Apparatus -** The experiments were conducted in eight operant chambers (Exterior: 17.8 cm x 15.2 cm x 18.4 cm; interior: 15.9 cm x 14.0 cm x 12.7 cm; base: 40.6 cm x 29.2 cm x 1.3 cm; Med Associates, St. Albans, VT, USA) placed in sound and light-attenuating enclosures fitted with fans for ventilation and reducing external noise. Each box was equipped with a central magazine (W=2.5 cm, H=2 cm) connected to an external food pellet dispenser to deliver 20 mg dustless precision food pellets (Bio-Serv Delivering Solutions, NJ, USA). Two levers (4×2 cm) were positioned on either side of the food magazine. A light emitting diode (LED) stimulus light was positioned centrally above each lever and a house light was located on the ceiling of each chamber. Each animal was trained and tested in the same operant chamber throughout the study The stimulus lights, house lights, and reinforcements were controlled and responses recorded by Med-PC software (Version 2.0 for DOS or Med-PC for Windows, Med Associates, Inc. VT, USA). Programs controlling the schedule of reinforcement were written using Med-State notation. **SM1b(ii) Operant training sessions – Training Phase -** Mice were trained in ORL task, with some modifications from (Abdul-Monim *et al*, 2006). Abdul-Monim et al used a fixed ratio (FR) of 1. We chose an FR of 10 in order to slightly increase the task difficulty. Initially, mice were trained to respond for food on an FR1 schedule of reinforcement, with both levers active and responses on either lever reinforced by delivery of a food pellet. Once stable responding was achieved, the FR was increased to 2, 5, and, finally, to 10. After stabilization of responding under an FR 10, RL training was initiated. The RL training sessions were conducted as follows: an animal was placed into an operant chamber and a pre-session of 10 min was started, during which all lights were extinguished, responses were not recorded, and had no programmed consequence. See Rajagopal et al., for our most recent ORL methodology (Rajagopal *et al*, 2017; Rajagopal *et al*, 2016b). Once the pre-session time elapsed, the house light and the stimulus light over the right-side lever were illuminated, signaling the start of a trial during which responses on only one lever was reinforced by food delivery (correct responses), whereas responses on the other lever did not result in food reinforcement. Once an FR 10 was completed on either lever, the animal either received reinforcement (if responding on the correct lever) or not. Next, all lights were extinguished for a 10-second inter-trial interval (ITI) during which responses had no consequence. After the 10-sec ITI elapsed, both lights were illuminated and a new trial started. The sessions consisted of 100 trials or 60 min had elapsed. The training phase continued until the subjects achieved 90% accuracy in responses on the reinforced lever on three consecutive days. After reaching criterion, the reinforcement contingencies were changed so that the opposite lever was activated and reinforced. Training resumed until the 3-day 90% criterion was reached. Reinforcement contingencies were “reversed” throughout the experiment as accuracy criteria were met. **SM1b(iii) Testing Phase -** Test drug or Veh was administered 30-45 minutes (depending on the pre-treatment time for the drug being investigated) prior to the initiation of test sessions. The same protocol described in the training phase was followed. After the pre-session elapsed, the house and stimulus lights were illuminated signaling the beginning of a 10-min period (the initial phase), during which the reinforcement contingency matched that of the previous day’s training session. At the conclusion of the initial phase, the house and stimulus lights were extinguished, and a 10-min time-out began. After the 10-min time-out elapsed, the house light was illuminated, and the next phase of the test session began (the reversal phase), during which the reinforcement contingency was reversed. The reversal phase consisted of trials across a 10-min period. The test session was terminated after the end of the 10-min reversal phase. The day following reversal test sessions, regular training sessions were conducted using the same reinforcement contingencies as in the reversal phase of the previous test session. Training sessions were conducted until the 3-day 90% accuracy criteria were again met, upon which another test session was conducted. This cycle of training and testing was continued throughout the experiment. The primary dependent measure of RL was the percent correct responses. Auxiliary dependent variables included number of correct trials, incorrect responses, latency to first response, number of incorrect trials, and rate of response.

**SM1b(iv) Data and Statistical Analysis:** Data for percent correct responding were calculated using the number of lever presses on the correct lever divided by the total number of presses multiplied by 100. The percent correct data was then analyzed by a one-way ANOVA followed by *post hoc* Bonferroni correction, to compare the effect of drug treatment of the scPCP-treated group. The total number of lever presses was calculated by adding the correct and incorrect presses together within the 20-min test session. This was used to assess whether drugs caused sedation or motor impairment.

**SM1c. Locomotor Activity (LMA):** Horizontal and vertical LMA were measured using a photo beam activity system (SD instruments, San Diego, CA, U.S.A.) which are armed with four pairs of infrared lights about 25 cm above the floor. Ambulatory, fine movements, and rearing movements were monitored for 120 min, at 15 min intervals, using a counting device programmed to count only when infrared light beams are interrupted consecutively. The animals were first habituated to the test box 30 min before the experimental protocol. For basal LMA, we evaluated the individual LMA to an acute injection of saline, which is considered as mild stress. In the drug treatment studies, e.g. PCP (10 mg/kg; i.p.) was given immediately after the 30 min habituation period and the LMA was recorded for a total of 120 min at eight 15 min intervals. **Statistical analysis:** Area under the curve (AUC) was calculated for the summation of 8 successive periods and one-way ANOVA was done followed by Bonferroni test when a significant effect was detected by ANOVA.

**SM1d. Microdialysis and Assay for neurotransmitters**

The method has been described in detail elsewhere (Huang et al 2015). Guide cannula (21 G) with dummy probes were placed and fixed by cranioplastic cement to mouse mPFC and dSTR. The stereotaxic coordinate of the implanted probe was A +2.0, L +0.5 (10˚ inclination), V –3.0 mm for the mPFC and A +1.0, L –1.5, V –4.5 mm for the dSTR, relative to the bregma (Paxinos and Franklin, 2004). Concentric-shaped dialysis probes (Synaptech Co., Marquette, MI, USA), with 2.0 mm of non-glued membrane surface (20,000 Da cut-off weight) were used for dialyzing. Two days after cannulation, dual dialysis probes were implanted into the mouse mPFC and dSTR under light anesthesia with isoflurance. Probes were implanted in the morning of dialysis day with perfusion at 1.0 μL/min of Dulbecco’s phosphate-buffered saline solution, 2 hours later, dialysate samples were collected every 30 minutes for measuring dialysate neurotransmitter concentration. After 4 samples were obtained as the baseline, drug or vehicle was administered. First injection was given at -30 min time point, and second injection was given at 0 min point as showed in the time response curves. The effect of the drug on neurotransmitter efflux was monitored for another 180 minutes. Samples were stored in -80ºC after collection until the assay. The details of the mass spectrometric/UPLC assay method are described elsewhere (Huang et al., 2014). The procedures applied in this experiment were approved by the Institutional Animal Care and Use Committee of Northwestern University, Chicago. N=8-10 per group.

Only results derived from healthy mice with correctly positioned dialysis probes were used in the data analysis. Mean pre-drug baseline levels (time points -90, -60, -30, and 0 minutes before drug or vehicle injection) were averaged and designated as 100%. Output levels for neurotransmitters were then expressed as a percentage of baselines. AUC (% of area under the curve, 0-180 min) were calculated and used for one way ANOVA-LSD test to determine the group differences (IBM SPSS statistics 20, IBM Co., NY, USA). Two-way ANOVA was used to determine the drug (pre-treatment*treatment) interactions. A probability of less than 0.05 was considered significant. All results are given as mean ± standard error of mean (SEM).

**SM1e. Schema of drug treatments for behavioral paradigms and in vivo microdialysis**


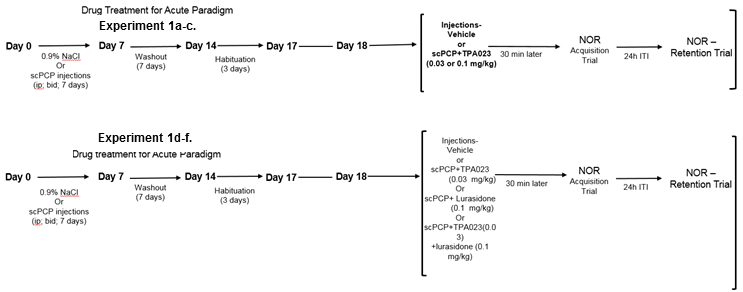


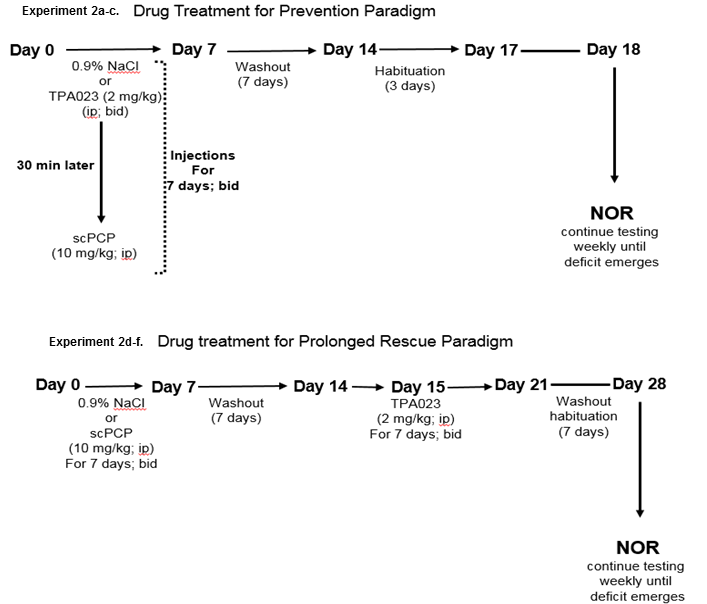


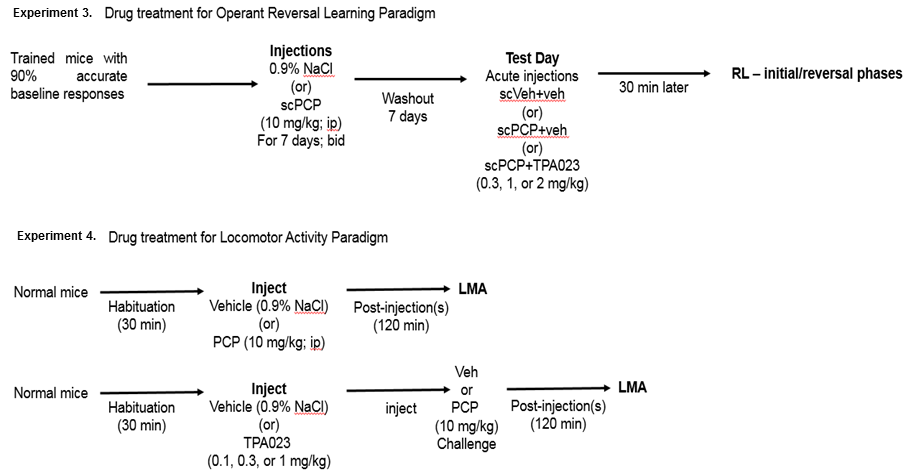


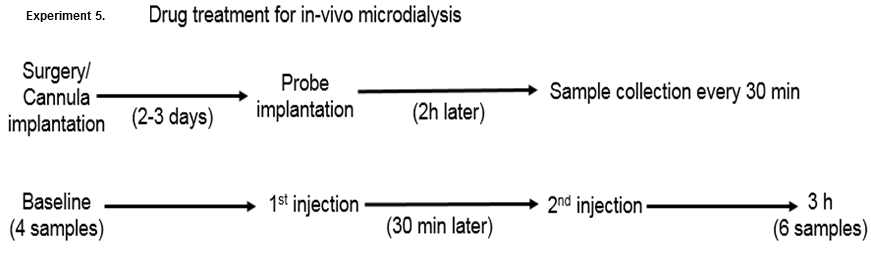


**ST1:** Auxiliary Measures for Reversal Learning experiment


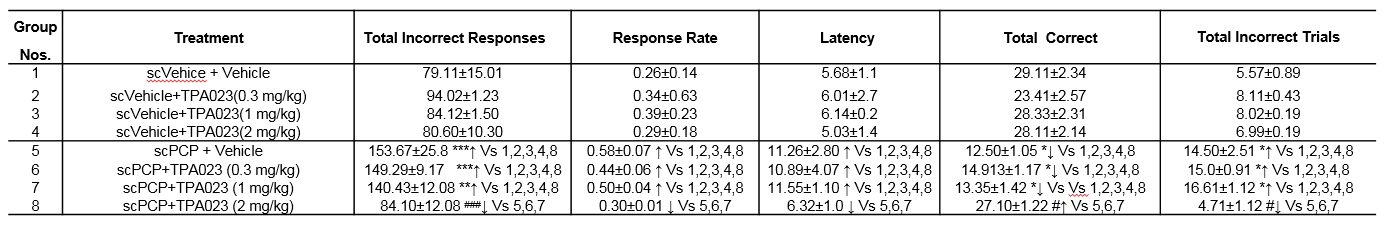


**ST1:** Mean ±SEM of auxiliary measures in all the groups in the reversal phase of the reversal learning task. Total Incorrect Responses: ***↑ - Significant increase (****p*<0.001) in total incorrect responses Vs (groups 1,2,3, 4, and 8 ( i.e. scVeh, scVeh+TPA023(0.3,1, and 2 mg/kg), and scPCP+TPA023 (2 mg/kg); Response Rate: ↑ - Non-significant trends of increase in response rate Vs groups 1, 2, 3,4, and 8; Latency: ↑ - Non-significant increase in latency vs groups 1,2,3,4,and 8; Total Correct: *↓ - Significant decrease (**p*<0.05) in total correct responses vs groups 1, 2,3, 4, and 8; # ↑ - Significant increase (#*p*<0.05) in total correct responses vs groups 5,6,and 7; Total Incorrect Trials: *↑ Significant increase (**p*<0.05) in total incorrect trials vs groups 1, 2, 3, 4, and 8; # ↓ Significant decrease (#*p*<0.05) in total incorrect trials vs 5,6, and 7.
